# Proteome analysis of bronchoalveolar lavage from calves infected with bovine respiratory syncytial virus—Insights in pathogenesis and perspectives for new treatments

**DOI:** 10.1371/journal.pone.0186594

**Published:** 2017-10-16

**Authors:** Sara Hägglund, Krister Blodörn, Katarina Näslund, Karin Vargmar, Sara Bergström Lind, Jia Mi, Mariluz Araínga, Sabine Riffault, Geraldine Taylor, John Pringle, Jean François Valarcher

**Affiliations:** 1 Swedish University of Agricultural Sciences, Host Pathogen Interaction Group, Dept. of Clinical Sciences, Uppsala, Sweden; 2 Swedish University of Agricultural Sciences, Section of Pathology, Department of Biomedical Sciences and Veterinary Public Health, Uppsala, Sweden; 3 Uppsala University, Science for Life Laboratory, Analytical Chemistry, Department of Chemistry-BMC, Uppsala, Sweden; 4 Binzhou Medical University, Medicine and Pharmarcy Research Center, Yantai, China; 5 University of Nebraska Medical Center (UNMC), Omaha, Nebraska, United States of America; 6 INRA, Unité de Virologie et Immunologie Moléculaires, Université Paris-Saclay, Jouy-en-Josas, France; 7 The Pirbright Institute Ash Road, Pirbright, Surrey, United Kingdom; Imperial College London, UNITED KINGDOM

## Abstract

Human and bovine respiratory syncytial viruses (HRSV/BRSV) are major causes of severe lower respiratory tract infections in children and calves, respectively. Shared epidemiological, clinical, pathological and genetic characteristics of these viruses make comparative research highly relevant. To characterise the host response against BRSV infection, bronchoalveolar lavage supernatant (BAL) from i) non-vaccinated, BRSV-infected ii) vaccinated, BRSV-infected and iii) non-infected calves was analysed by tandem mass spectrometry. Proteins were semi-quantified and protein expression was validated by immunoblotting. Correlations between selected proteins and pathology, clinical signs and virus shedding were investigated. Calves with BRSV-induced disease had increased total protein concentrations and a decreased number of proteins identified in BAL. The protein profile was characterised by neutrophil activation and a reduction in identified antioxidant enzymes. The presence of neutrophils in alveolar septa, the expression level of neutrophil-related or antioxidant proteins and LZTFL1 correlated significantly with disease. Citrullinated histone 3, an indicator of extracellular traps (ETs), was only detected in non-vaccinated, BRSV-infected animals. By bringing disequilibrium in the release and detoxification of reactive oxygen species, generating ETs and causing elastine degradation, exaggerated neutrophil responses might exacerbate RSV-induced disease. Neutrophil-mitigating or antioxidant treatments should be further explored.

## Introduction

Human and bovine respiratory syncytial viruses (HRSV and BRSV) are host-specific but genetically highly similar and induce comparable pathology in humans and cattle [1]. It is suspected that the clinical signs induced by RSV partly derive from inflammatory responses. In humans, severe RSV-induced inflammation was shown to additionally have long-term consequences including airway hyper-reactivity during childhood (reviewed by [2]). As recently reviewed by Dapat and Oshitani (2016), proteomic data based on mass spectrometry are sparse from *in vivo* RSV infections [3], but does exist on nasal aspirates from HRSV-infected children, lung tissue from HRSV-infected rats, and from mice with vaccine-induced enhanced HRSV disease [4–6]. Overall, proteins were separated by gel-electrophoresis. To complement these approaches, by using an experimental model of RSV infection in a natural host that reproduces severe clinical signs of disease [7] and by analysing the proteomic profile of samples collected from the lower respiratory tract at precise times post infection, we assumed that we could obtain new information about RSV pathogenesis. Thereby, targets for new treatments could potentially be identified. We used label free relative quantification by liquid chromatography coupled to tandem mass spectrometry (LC-MS/MS), the shotgun method [8], to investigate the proteomes of bronchoalveolar lavage supernatants (BAL) obtained from calves at the peak of clinical signs and analysed data in relation to pathology, clinical score, and virus shedding.

## Material and methods

### Samples, animals and experimental design

Bronchoalveolar lavage (BAL) was collected post mortem, as previously described [9], from the left lung of 15 conventionally-reared, 2–4 months old calves of the Swedish red and white and Swedish Holstein breeds that had been infected with BRSV by aerosol in two independent studies (Table 1).

**Table 1:** Animals and experimental design.

Study	No. calves	Treatment	BRSV strain	Virology	% lung lesions	Designation	Mean BAL protein conc. (μg/μl)	Mean no. proteins identified
I	5	Non-vaccinated	9402022	+++	15.7 ± 14.5	Susceptible_I	0.49	394
	5	BRSV-ISCOM-vaccinated	9402022	±	3.8 ± 2.9	Vaccinated	0.19	547
II	5	Non-vaccinated	Snook	+++	48 ± 12.1	Susceptible_II	0.79	159
	5	None	None	-	0	Non-infected	0.37	296

The allocation to groups, the extent of macroscopic lung lesions, the clinical scores (prior and post infection) and the quantity of virus shed in nasal secretions in these calves has been documented in detail elsewhere [9, 10]. The clinical scoring system is available as supplemental data (S1 Table). From these animals tissue samples from the right cranial lobe, if present from an area of consolidation, were processed for routine diagnostic evaluation with hematoxylin/eosin-staining. The degree and characteristics of the inflammatory response was assessed in a blinded manner by a diagnostic pathologist. In case of histopathological lesions, including infiltration by inflammatory cells, they were subjectively graded mild, moderate to severe, e.g. the degree of neutrophils in alveolar septa was scored (in text represented as score 0–3, score 0 negative and 3 severe). BAL was also collected from 5 uninfected, clinically healthy calves of the same breeds and approximate age at slaughter. Calves with no or minimal macroscopic lung lesions were selected for this purpose.

Fresh BALs were kept on ice, filtered over sterile gauze, centrifuged at 200 x *g* for 10 minutes and the supernatants were stored at -80°C, before analyses in two separate experiments:

In experiment I, the BAL originated from calves that had been infected with BRSV (strain no. 9402022, kindly provided by Pr. L.E. Larsen, DTU, Denmark), 6 days previously, and were either a) non-vaccinated and showed clinical signs of disease and shed high quantities of virus (n = 5, called susceptible_I), or b) BRSV-ISCOM-vaccinated and showed little or no clinical signs of disease and shed little or no virus (n = 5, called vaccinated) (Table 1) [9]. As described in detail previously [11], this vaccine consisted of pleomorphic nanoparticles that contained purified, solubilized proteins from BRSV-infected cell culture, as well as lipids and Quillaja saponin.

In experiment II, the BAL originated from calves that had either been infected with BRSV (Snook strain), 7 days previously, and shed high quantities of virus, showed severe clinical signs of disease and had extensive macroscopic lung lesions (n = 5, called susceptible_II) [10], or were non-infected and healthy, at slaughter (n = 5, called non-infected) (Table 1). The clinical signs and pathology in the susceptible animals were more severe in experiment II than in experiment I. In experiment II, five calves showed marked to severe signs of illness, *versus* two calves in experiment I. BRSV had been isolated in cell culture by using BAL cells (*i*.*e*. not the supernatant), from all susceptible calves and one vaccinated calf, in experiment I and II [9, 10].

### Animal care

Both experiments were carried out in compliance with the E.U. Directive 86/609, and approved by the Ethical Committee of the district court of Uppsala, Sweden (Refs. no. C68/10, C330/11). The health and behaviour of all animals were monitored at least twice daily throughout the experiments (6 and 7 weeks) and at least three times daily from the day of challenge to the day of euthanasia (day 6 or 7 post infection). The humane endpoint, which was used as criterion for euthanasia, was defined as follows: i) abdominal or forced breathing, or respiratory rate exceeding 100 min^-1^, in combination with severely depressed general condition, or ii) anorexia for more than 24 hours, or iii) rectal temperature exceeding 41°C for more than 36 hours. One calf in experiment II reached the humane endpoint on PID7 and was euthanized immediately after the clinical examination. None of the calves died before reaching the endpoint.

To minimize suffering and distress of the animals, the bedding was kept clean and thick and the air and food quality was optimal. The calves were fed hay and water *ad libitum*, and pellets and milk replacer twice daily. They were frequently handled, to reduce stress at sample collection. Clinical examinations and sample collections were performed by trained veterinarians, certified by individual licenses to perform animal experiments. The calves were euthanised by an overdose of general anesthesia (5 mg/kg ketamine and 15 mg/kg pentobarbital sodium) followed by exsanguination. The calves with the most severe clinical signs were euthanized first.

### Liquid chromatography tandem mass spectrometry (LC-MS/MS) and data analyses

BAL supernatants (hereafter called BAL) containing 20 μg total protein, determined using the Bradford assay (BioRad), were diluted to contain 50 mM ammonium bicarbonate. The samples were reduced with dithiothreitol (DTT) and alkylated with iodoacetamide. Trypsin was added in a trypsin:protein ratio of 1:20 and digestion was performed overnight. Thereafter the samples were purified by Pierce C18 Spin Columns (Thermo Scientific), dried and resolved in 60μL 0.1% formic acid, and analysed by tandem mass spectrometry. Analyses were performed in a blind manner using peptide separation by reversed phase liquid chromatography and an QExactive Plus Orbitrap mass spectrometer (Thermo Fisher Scientific, Bremen, Germany) equipped with a nano electrospray ion source, as detailed in [12], with the following modifications: Protein identification was performed against a FASTA database containing proteins from *Bos Taurus* extracted from the SwissProt database (release Oct 2016, 44366 entries). Search parameters included a maximum of 4.5 ppm and 20 ppm error tolerances for the survey scan and MS/MS analysis, respectively. For protein identification, only peptides with a minimum of 7 amino acids were considered. A total label free intensity analysis was performed for each individual sample. Pathway analyses were carried out in Reactome v59 [13], (www.reactome.org). Proteins were classified as detected at calf treatment group level if identified in at least 4 out of 5 calves and venn diagrams were designed using the Interactivenn software [14], (www.interactivenn.net). Protein functions were analysed in Panther v11.1 [15], (www.pantherdb.org/).

### Immunoblotting

Dotblots and Western blots were carried out as described previously [11], using polyclonal rabbit antibodies against myeloperoxidase or histone H3 citrulline 2+8+17 peptide (ab14323 and ab5103, Abcam,) and polyclonal HRP-conjugated sheep antibodies against rabbit IgG (Star 54, Bio-Rad). Controls using BAL antigen and the secondary antibodies alone were included. Protein bands were quantified using Image Lab 5.2.1 (BioRad).

### Bacterial analyses

All BRSV-infected animals had been treated with penicillin prior to BRSV-infection. The presence of bacteria in the lungs was determined by aerobic culture of fresh BAL from BRSV-infected calves on bovine blood agar. Since all animals were conventionally reared, analyses of DNA specific for the bacterial opportunists *Pasteurella multocida*, *Mannheimia haemolytica*, *Histophilus somni* and *Mycoplasma bovis* were additionally performed by TaqMan® qPCR (LSI VetMAX™ Screening Pack, Life Technologies) on BAL from all animals but two (vaccinated calves b and d), due to lack of material from these individuals. The assay was performed according to the manufacurer’s instructions.

### Statistical analyses

Statistical analyses were carried out in Minitab® software, version 17. For comparisons of label free quantification (LFQ) of intensities for relative protein expression level in BAL, a two-tailed student’s T-test was used, with assumed unequal variance. For correlations, the Spearman's rank and Pearson product moment correlation coefficients were used for data with non-normal and normal distribution, respectively (determined using the Anderson-Darling test). Samples from all animals were included in all analyses, if not otherwise specified.

## Results

### BRSV-infection increased extracellular protein levels and decreased the number of proteins identified in the lumen of the lower respiratory tract

In experiment I, 6 days post BRSV-infection (DPI), the total protein concentration in BAL was significantly higher in susceptible_I calves than in vaccinated animals which were protected against BRSV infection (mean 0.49 μg/μL (0.20–0.71) and 0.19 μg/μL (0.12–0.46), respectively; p = 0.04) (Table 1). Similarly, in experiment II, the total protein concentration was higher in BAL of susceptible_II calves 7 DPI than in that of non-infected animals, although this was not statistically significant (mean 0.79 μg/μL (0.07–1.34) and 0.37 μg/μL (0.10–0.68), respectively; p = 0.15) (Table 1).

The number of proteins identified by LC-MS/MS per BAL was significantly lower in the susceptible_II calves compared to non-infected animals (mean 159 (64–240) and 296 (124–349), respectively; p = 0.03) and tended to be lower in the susceptible_I calves than in the vaccinated animals (mean 394 (322–504) and 547 (249–702), respectively; p = 0.11) (Table 1).

### BRSV-infection increased the expression of 96 proteins involved in several biological processes

**Fig 1:**
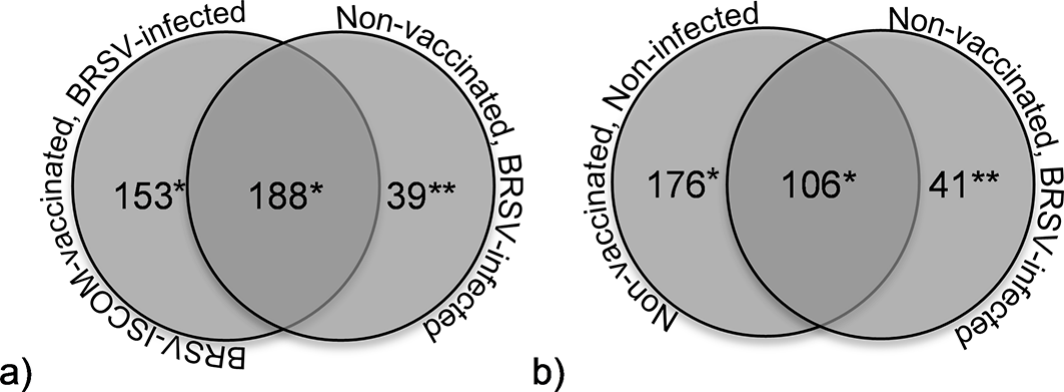
Bronchoalveolar proteomes of calves. Proteins were identified by LC-MS/MS in bronchoalveolar lavage of calves and were classified as detected at group level if identified in at least 4 out of 5 calves per group. The groups consisted of non-vaccinated, BRSV-infected calves with clinical signs of disease and high levels of virus shedding (a and b, right circles), or ISCOM-vaccinated, BRSV-infected calves with no or little clinical signs of disease and no or low levels of virus shedding (a, experiment I, left circle), or non-vaccinated, non-infected calves (b, experiment II, left circle). Protein names are provided as supplemental data (*) or in Table 1 (**).

In addition to differences in the total protein concentration and number of proteins identified, the pattern of proteins identified in BAL differed between susceptible calves with high virus shedding and vaccinated calves with very little or no virus shedding, or non-infected calves. Qualitatively, 39 and 41 proteins were uniquely detected in susceptible calves, in experiment I and II, respectively (Fig 1A and 1B, right side of right circles; Table 2, Qualitative).

**Table 2:** Proteins associated with BRSV-induced disease.

Qualitative: Proteins classified as uniquely detected in non-vaccinated, BRSV-infected, susceptible calves and not in controls^a^^,^^b^		Quantitative: Proteins identified at significantly higher expression levels in non-vaccinated, BRSV-infected, susceptible calves compared to controls^a^^,^^c^	
Calf experiment I	Calf experiment II	Calf experiment I	Calf experiment II
Actin, alpha cardiac muscle 1		Actin, alpha cardiac muscle 1*	
Actin-related protein 2/3 complex subunit 5		Actin-related protein 2/3 complex subunit 5*	
		Adenylyl cyclase-associated protein**	
	Alpha-1B-glycoprotein		Alpha-1B-glycoprotein*
		Annexin A1 *	
Azurocidin	Azurocidin	Azurocidin*	
			Beta-2-microglobulin*
Beta-defensin (2/8/10)	Beta-defensin (2/8/10/13)	Beta-defensin (10*)	Beta-defensin (2**/8*/10*/12**)
BRSV (M/M2-1/N /P)		BRSV(M*/M2-1*/N* /P**)	
Calponin-2			
Cathelicidin (4/5/7)	Cathelicidin (5)	Cathelicidin (1***/3*/4*/5**)	Cathelicidin (1**/2*/3*/4**/5**/6*/7**)
Cathepsin (G/L2)	Cathepsin (G like)	Cathepsin (G**/L2*)	Cathepsin (G**)
	CD14		CD14**
	CD177	CD177**	
	CD44		
	Complement factor properdin	Complement factor properdin**	
		Coronin-1A**	
		Cystatin-C**	
	ECM1 protein		
EF-hand domain-containing protein D2		EF-hand domain-containing protein D2**	
Elastase	Elastase	Elastase*	Elastase*
Folate receptor alpha	Folate receptor alpha	Folate receptor alpha*	
		Fructose-bisphosphate aldolase*	
		Glucose-6-phosphate isomerase*	
		Glyceraldehyde-3-phosphate dehydrogenase*	
Granulin	Granulin	Granulin**	Granulin**
		Haptoglobin*	Haptoglobin*
Hematopoietic cell-specific Lyn substrate 1	Hematopoietic cell-specific Lyn substrate 1		Hematopoietic cell-specific Lyn substrate 1*
		High mobility group protein B2*	
Histone (H2A/H2AB/H2B)	Histone (H1e/H1.1/H1.2/H1.3/H2A.J/H2B 1/H2B 1-K/ H3.2)	Histone (H1.3*/H2A*/H2A 2-C*/H2B*/H3.1*/H4*)	Histone (H1e*/H1.1*/H1.2*/ H2AC*/H2AJ*)
	Ig lambda chain V-I region BL2-like		Ig lambda chain V-I region BL2-like**
	Ig like V-set		Ig like V-set*
	Inter-alpha-trypsin inhibitor heavy chain H4		
		Lactotransferrin**	Lactotransferrin*
	Lingual antimicrobial peptide		Lingual antimicrobial peptide*
L-serine dehydratase/L-threonine deaminase	L-serine dehydratase/L-threonine deaminase	L-serine dehydratase/L-threonine deaminase**	
Lymphocyte cytosolic protein 1		Lymphocyte cytosolic protein 1*	
Lymphocyte-specific protein 1	Lymphocyte-specific protein 1		
Macrophage migration inhibitory factor			
		Matrix metalloproteinase-9***	
MSLN protein			
	Myeloperoxidase	Myeloperoxidase**	Myeloperoxidase*
Neutrophilic granule protein-like		Neutrophilic granule protein-like*	
Nucleobindin 2		Nucleobindin 2**	
		Olfactomedin 4**	
Pentraxin-related protein PTX3		Pentraxin-related protein PTX3**	
		Peptidoglycan recognition protein 1**	Peptidoglycan recognition protein 1*
	Primary amine oxidase, liver isozyme		
Protein FAM49B			
Protein S100-A9	Protein S100-A9	Protein S100 (A8**/A9*)	
	Proteinase 3	Proteinase 3**	
		Ras-related C3 botulinum toxin substrate 2*	
	Regakine-1		
	Resistin	Resistin*	Resistin**
Rho GDP-dissociation inhibitor 2		Rho GDP-dissociation inhibitor 2*	
	Secretoglobin family 1D member		
SERPINB4 protein		SERPINB4 protein**	
Serum amyloid A protein (3.2)	Serum amyloid A protein (1/3.2)		Serum amyloid A protein (1*/3.2**)
Spleen trypsin inhibitor I		Spleen trypsin inhibitor I***	
		Sulfhydryl oxidase*	
	Transaldolase	Transaldolase***	
Uteroglobin			
		Transcobalamin 1***	
		Transketolase**	
			Uncharacterised (G5E604^d^)**
			Uncharacterised (ENSEMBL:ENSBTAP00000014147^d^)*

^a^Proteins were detected in bronchoalveolar lavage from non-infected and/or BRSV-infected calves by LC-MS/MS and semi-quantified by label free analysis. Grey highlight; proteins associated with BRSV-induced disease, in both animal experiments I and II

^b^Uniquely detected at group level in non-vaccinated, BRSV-infected, susceptible calves (i.e. detected in at least 4 out of 5 non-vaccinated, BRSV-infected, susceptible calves and in less than 4 ISCOM-vaccinated, BRSV-infected, protected calves (Experiment I) or non-infected calves (Experiment II)).

^c^Detected at significantly higher expression levels in non-vaccinated, BRSV-infected, susceptible calves compared to in ISCOM-vaccinated, BRSV-infected, protected calves (Experiment I) or non-infected calves (Experiment II)).

^d^Protein identification number, uncharacterized protein, no gene identified

Statistically significant differences are indicated by asterisks; p≤0.05(*); p≤0.01 (**); p≤0.001 (***).

Quantitatively, 57 and 35 proteins were expressed at significantly higher levels in BRSV-infected, susceptible calves compared to vaccinated or non-infected calves in experiments I and II, respectively (Table 2, Quantitative).

In total, 96 different proteins were associated with BRSV-disease, either qualitatively or quantitatively (Table 2), 44 of which were common between experiment I and II, or belonged to protein families in which one or several proteins were common between the experiments (Table 2, grey highlight). The common proteins associated with BRSV infection were related to the following cells and processes:

- Neutrophil activation and chemotaxis, including neutrophil extracellular trap (NET) formation [16–21]: azurocidin, beta defensins, cathelicidins, cathepsins, elastase, granulin, hematopoietic cell-specific Lyn-substrate 1, histones, lactotransferrin, lymphocyte-specific protein 1, myeloperoxidase, peptidoglycan recognition protein, proteins S100-A9 and resistin.
- Epithelial cells and epithelial-derived responses [16, 22, 23]: beta defensins, cathelicidins, folate receptor alpha.
- Lymphocyte and natural killer cell activation and chemotaxis [24, 25]: hematopoietic cell-specific Lyn-substrate, lymphocyte-specific protein 1.
- Macrophage activation [26]: beta defensins, cathelicidins, resistin.
- Systemic acute-phase responses [27]: serum amyloid A, haptoglobin.
- Cell necrosis and/or apoptosis [28]: histones.
- Gluconeogeneis [29]: L-serine dehydratase/L-threonine deaminase.

BRSV proteins were identified in 5/5 susceptible_I calves and 2/5 susceptible_II calves but not in any of the vaccinated or non-infected calves. The immunoglobulin J chain was detected in all calves in both experiments and the heavy constant μ chain was detected in all calves in experiment II, but the expression levels of these proteins did not in differ significantly between groups.

### Less antioxidant proteins were detected following BRSV-infection

Qualitatively, 153 and 176 proteins were uniquely detected in vaccinated and non-infected calves, respectively (Fig 1A and 1B, left side of left circles, supplemental data S2 Table), and quantitatively, 95 and 101 proteins were expressed at significantly lower levels in BRSV-infected, susceptible calves compared to vaccinated and non-infected calves, respectively (supplemental data S3 Table). Thirty-eight of the proteins identified at lower expression levels in the animals with BRSV disease were common between the two experiments and were submitted to Panther for classification [15]. In total, 36 proteins were classified and the major protein class was oxidoreductase (n = 9), followed by cytoskeletal proteins (n = 5), hydrolases (n = 4) and chaperone proteins (n = 4). The remaining nine protein classes contained 1–3 proteins.

### Pathway analysis confirmed increased neutrophil degranulation and reduced detoxification of reactive oxygen species associated with BRSV infection

**Fig 2:**
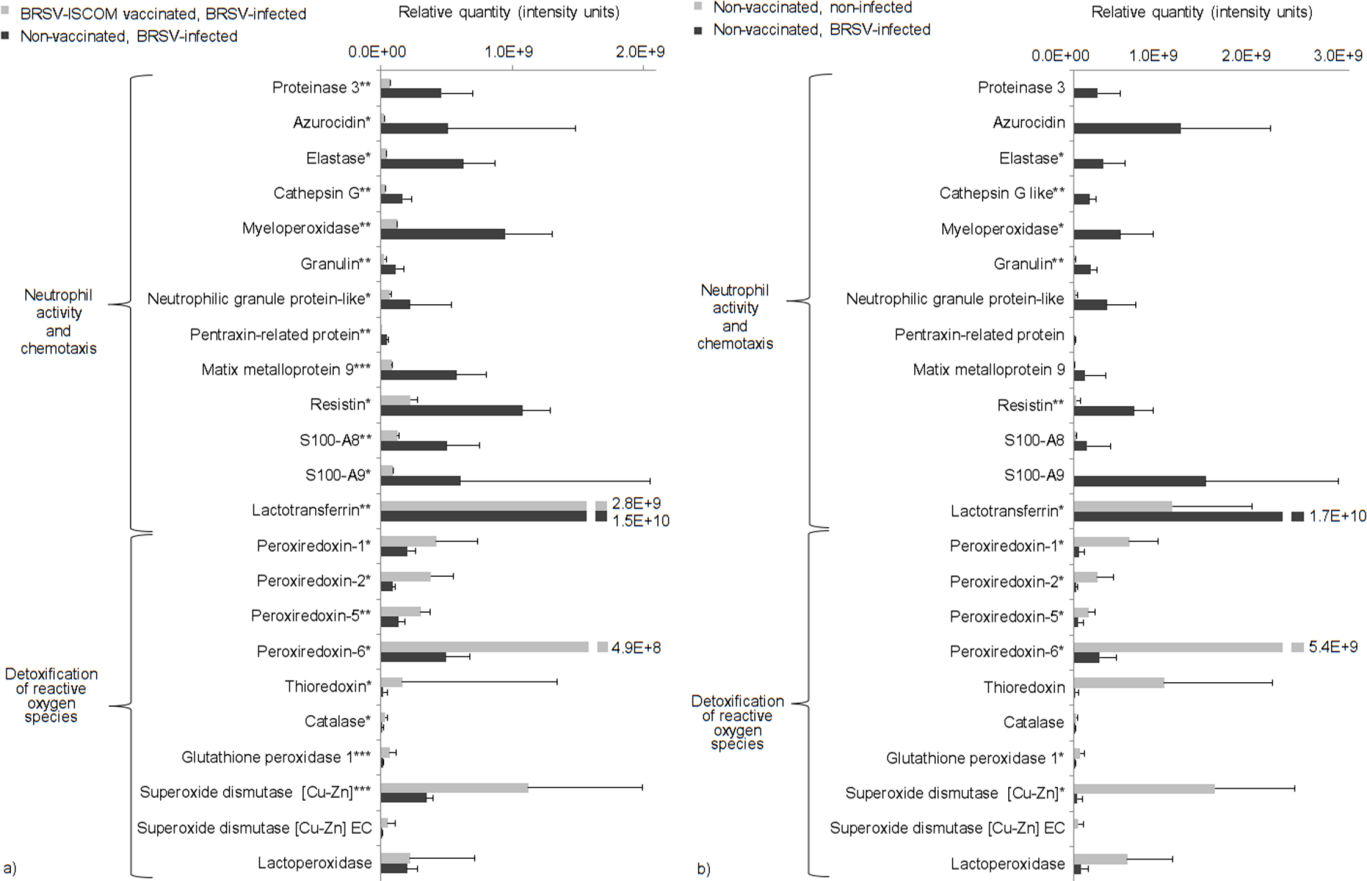
Semi-quantification of selected proteins involved in processes that were affected by BRSV-infection. Proteins were detected in bronchoalveolar lavage from non-vaccinated, BRSV-infected calves with clinical signs of disease and high levels of virus shedding (black bars) and/or in BRSV-ISCOM-vaccinated, BRSV-infected calves with no or little clinical signs of disease and no or low levels of virus shedding (a, experiment I, grey bars), and/or non-vaccinated, non-infected calves (b, experiment II, grey bars). Proteins were identified by LC-MS/MS and semi-quantified by label-free analysis. Statistically significant differences are indicated by asterisks; p≤0.05(*); p≤0.01 (**); p≤0.001 (***). Proteins were selected based on being related to neutrophil activation and chemotaxis or detoxification of reactive oxygen species: biological processes identified by protein pathway analysis.

The four quantitative datasets, containing the 57 and 35 proteins identified at statistically significantly higher expression levels in BRSV-infected, susceptible calves (Table 2, Quantitative), as well as the 95 and 101 proteins identified at statistically significantly lower expression levels in these animals compared to those in their vaccinated or non-infected controls, in experiment I and II, respectively (S3 Table), were submitted to Reactome [13]. The numbers of proteins that were recognized and mapped were as follows: 29/57 (higher, experiment I), 10/35 (higher, experiment II), 56/95 (lower, experiment I) and 52/101 (lower, experiment II). The bovine cathelicidins 1–7, which were increased in animals with BRSV disease in experiments I and II (Table 2) were among the proteins not recognized by Reactome. Nevertheless, in both experiments, the pathway identified with the highest probability to be activated during BRSV infection was neutrophil degranulation. The pathway identified with the highest probability to be downregulated, inactive or not identified during BRSV infection was detoxification of reactive oxygen species. The relative expression levels of proteins included in these pathways followed the same pattern, which is illustrated in Fig 2 (see black bars for BRSV-infected, susceptible calves).

**Fig 3:**
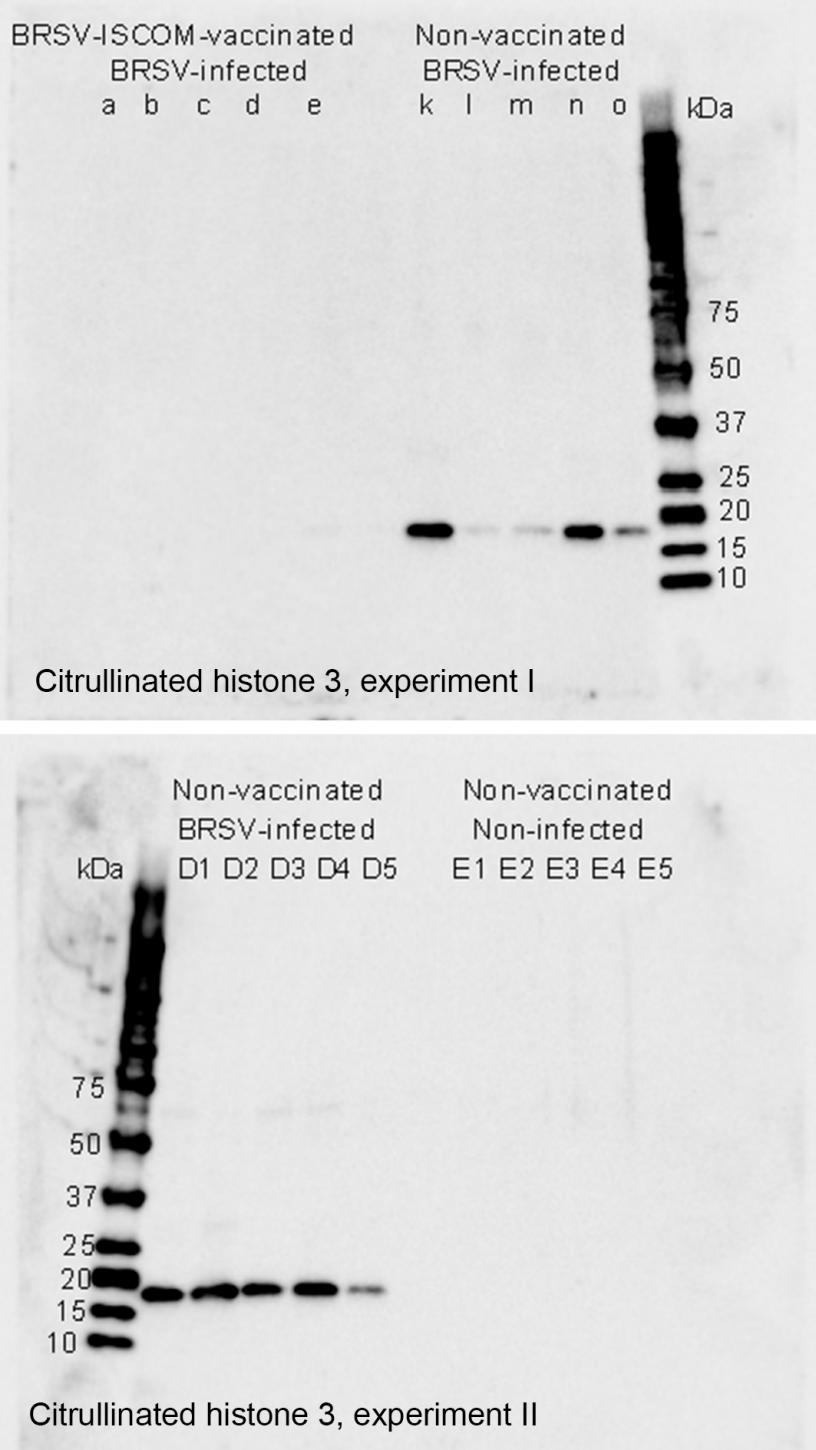
Association of citrullinated histone 3 with BRSV disease. Detection of citrullinated histone 3 by Western blot in bronchoalveolar lavage from BRSV-infected calves with clinical signs of disease and high levels of virus shedding (k-o and D1-D5) compared to that in BRSV-ISCOM-vaccinated, BRSV-infected calves with no or little clinical signs of disease and no or low levels of virus shedding (a-e, Experiment I), or non-vaccinated, non-infected calves (E1-E5, Experiment II).

**Fig 4:**
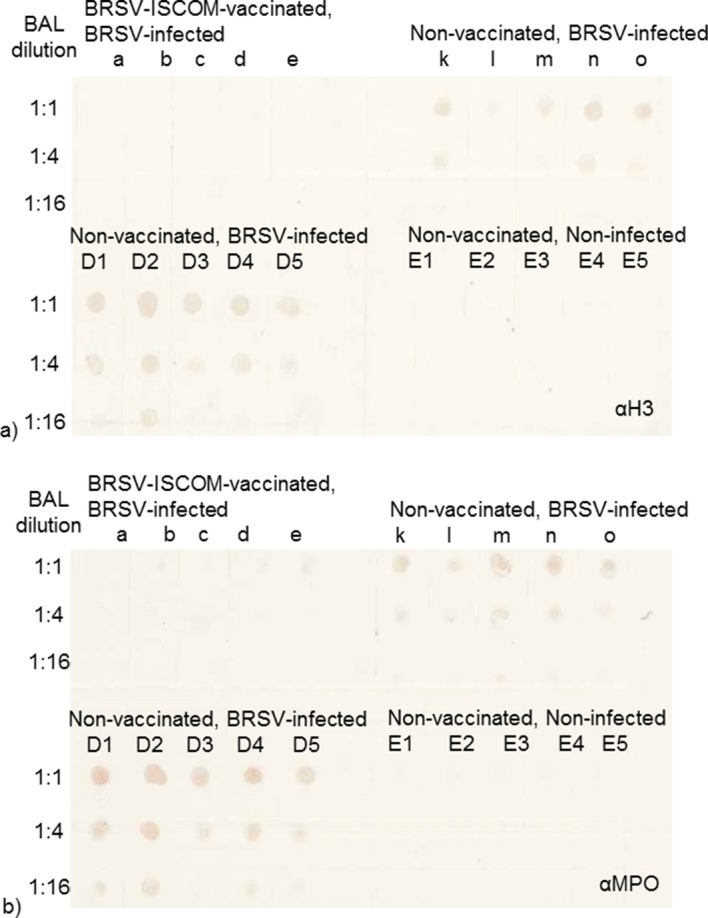
Detection of citrullinated histone 3 and myeloperoxidase in serially diluted BAL. Detection of citrullinated histone 3 (a) and myeloperoxidase (b) by dotblot in bronchoalveolar lavage (BAL) from BRSV-infected calves with clinical signs of disease and high levels of virus shedding (k-o and D1-D5) compared to that in BRSV-ISCOM-vaccinated, BRSV-infected calves with no or little clinical signs of disease and no or low levels of virus shedding (a-e, Experiment I), or non-vaccinated, non-infected calves (E1-E5, Experiment II). The proteins were selected based on being present in neutrophil extracellular traps, incriminated to be important in the pathogenesis of RSV [30].

The presence of proteins related to neutrophil activity was confirmed by immunoblotting on serially diluted BAL. Citrullinated histone 3 and myeloperoxidase were detected in BAL from all or several BRSV-infceted, susceptible_I and susceptible_II calves, but not in their vaccinated or non-infected controls (Figs 3 and 4). Citrullinated histone 3, detected by western blot at 17 kDa, was semi-quantified within each of the two experiments. When compared to k (experiment I), or D1 (experiment II) the relative quantity was estimated to 100%, 5%, 8%, 82%, 20% (calves k-o) and 100%, 87%, 68%, 94%, 20% (calves D1-D5).

### The level of neutrophil activation correlated with the degree of pathology, clinical signs and virus shedding

**Fig 5:**
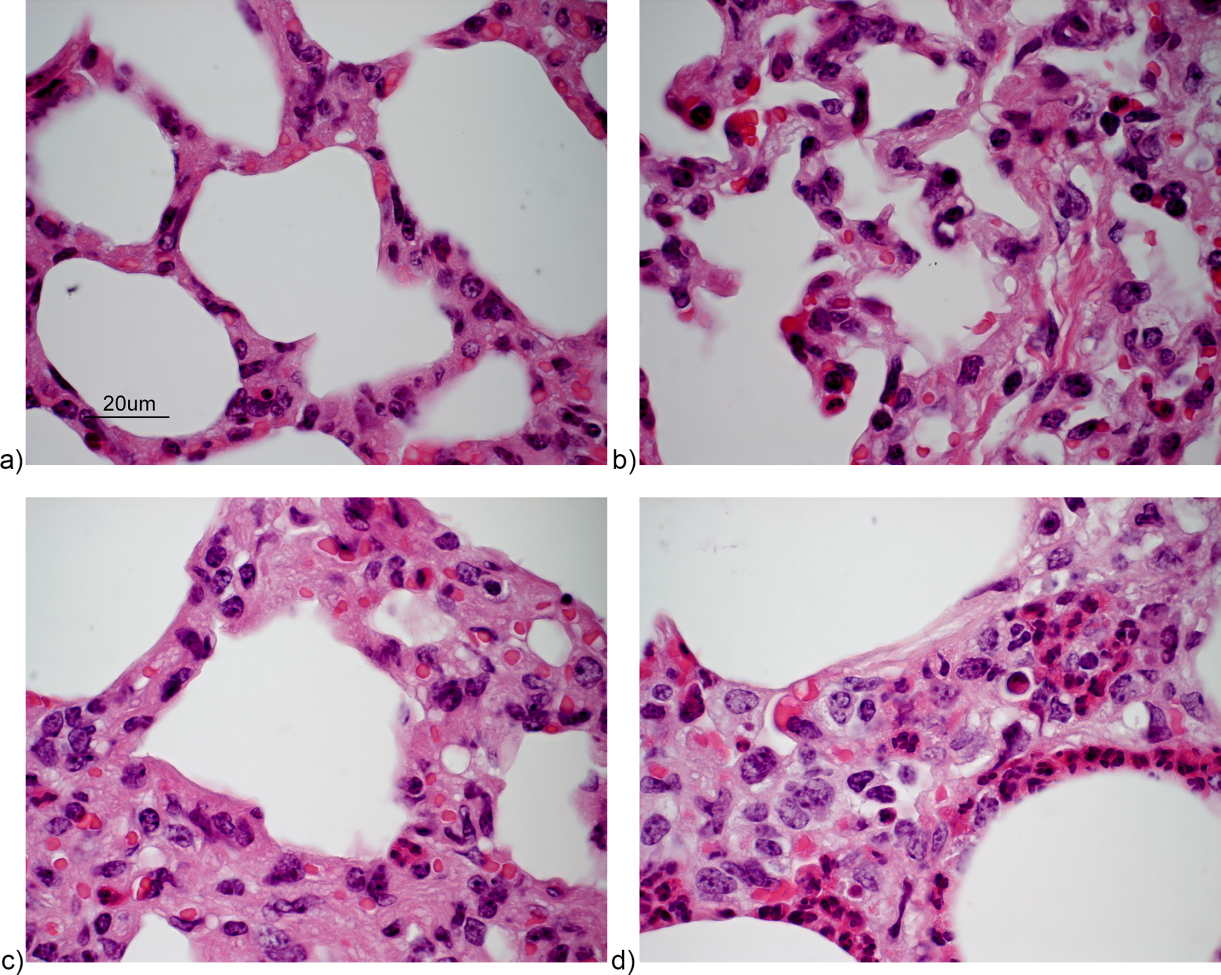
Scores of neutrophilic infiltration of alveolar septa in BRSV-infected calves. HE stained sections. a) score 0 (vaccinated calf a, experiment I), b) score 1 (vaccinated calf b, experiment I), c) score 2 (non-vaccinated, BRSV-infected calf l, experiment I) and d) score 3 (non-vaccinated, BRSV infected calf D5, experiment II). Lesions are examples of neutrophil scores and not representative for the overall inflammation regarding exudate and consolidation.

To determine if high neutrophil activity constituted a protective immune response in infected animals or, may have contributed to clinical signs of disease, the degree of pulmonary neutrophil-infiltration was evaluated in relation to BRSV-induced disease in experiment I, because the calves in this experiment had varying levels of disease and neutrophil scores [9]. The susceptible calves in experiment II were all very severely affected and all had the highest neutrophil score (for examples of neutrophil scoring, see Fig 5).

In experiment I, the neutrophil score in alveolar septa ranged from 1–3 (median 2) in BRSV-infected, susceptible_I calves and between 0–1 (median 1) in vaccinated calves. Overall, there was a significant correlation between severity of clinical signs, pathology and viral load and the presence of neutrophils in alveolar septa (Spearmans rho 0.71, p = 0.02, for extent of lung lesions; 0.63, p = 0.05 for clinical score; and 0.87, p = 0.001 for virus shedding). Two of these correlations were also statistically significant within just the group of five BRSV-infected, susceptible_I animals, *i*.*e*. those for lung lesions (Pearson’s r: 0.89, p = 0.04) and virus shedding (Pearson’s r: 0.93, p = 0.02), but not for clinical score (Pearson’s r: 0.72, p = 0.17).

To confirm these data and to validate the mass spectrometric results for neutrophil-related proteins, the relative expression level of selected proteins detected by LC-MS/MS in BAL was analysed in relation to the degree of BRSV-induced gross pathology, clinical score, virus shedding and degree of neutrophils in alveolar septa. A significant positive correlation between these parameters and the relative expression level of elastase, myeloperoxidase, resisitin and S100-A9 was observed (Table 3).

**Table 3:** Correlations (Spearman’s rho) between relative expression level of selected proteins in bronchoalveolar lavage from calves six days post BRSV infection, BRSV-induced disease and presence of neutrophils in alveolar septa.

Protein	Lung lesion exent^a^^,^^b^	Clinical score^a^^,^^c^	Virus shedding^a^^,^^d^	Neutrophils in septa^e^
Elastase	0.78, p = 0.007	0.77, p = 0.009	0.80, p = 0.006	0.70, p = 0.025
Myeloperoxidase	0.69, p = 0.028	0.67, p = 0.036	0.79, p = 0.006	0.80, p = 0.005
Resistin	0.78, p = 0.008	0.85, p = 0.002	0.88, p = 0.001	0.89, p = 0.001
S100-A9	0.75, p = 0.013	0.85, p = 0.002	0.88, p = 0.001	0.83, p = 0.003
Superoxide dismutase	-0.70, p = 0.024	-0.79, p = 0.007	-0.82, p = 0.004	-0.78, p = 0.009
Gluthathione peroxidase	n.s.	-0.64, p = 0.048	-0.74, p = 0.014	-0.73, p = 0.018
Peroxiredoxin-6	n.s.	-0.69, p = 0.027	-0.74, p = 0.015	n.s.
Leucine Zipper TFL1	n.s.	-0.76, p = 0.010	-0.71, p = 0.021	n.s.

^a^Proteins were identified and semi-quantified by LC-MS/MS including label free analysis, in bronchoalveolar lavage (BAL) from non-vaccinated, BRSV-infected calves with clinical signs of disease and high levels of virus shedding (n = 5) and in BRSV-ISCOM-vaccinated, BRSV-infected animals with no or little clinical signs of disease and no or low levels of virus shedding (n = 5).

^b^Correlation of protein with extent of pathologic lung lesions at the day of BAL collection, post infection day (PID) 6

^c^Correlation of protein with sum of clinical scores PID 6

^d^Correlation of protein with quantity of BRSV-RNA detected in nasal swabs PID 6

^e^Correlation of protein with extent of neutrophils in alveolar septa on one histological section from the right cranial lobe (from areas with macroscopic consolidation, if present) on PID 6. The presence of neutrophils was graded between 0 and 3.

n.s; not satistically significant (p>0.05)

Resistin and S100-A9 correlated best with BRSV-induced clinical signs, virus shedding and neutrophils, whereas elastase and resistin correlated best with lung lesions (Table 3). In contrast, a significant negative correlation was observed between the disease parameters and several antioxidant proteins or the leucine zipper transcription factor-like protein 1 (Table 3). A significant negative correlation was also observed between the relative expression level of each of the antioxidant proteins and each of the neutrophil-related proteins listed in Table 3 (p<0.05 for all comparisons, Spearman’s rho, supplemental data S4 Table). In summary, high levels of resistin, S100-A9, and elastase and low levels of antioxidant proteins and leucine zipper transcription factor-like protein 1 correlated with the severity of BRSV disease.

The inflammation was characterised by mild to severe broncho-interstitial or interstitial pneumonia, typical of BRSV, in all BRSV-infected calves. Despite negative bacterial cultures, DNA from *Pasteurella multocida* was detected in BAL from 5 out of 8 tested calves in experiment I (calf a (ct-value 33), k (ct-value 26), l (ct-value 35), n (ct-value 40) and o (ct-value 29)). DNA from *Pasteurella multocida* was not detected in BAL from any of the calves in experiment II (D1-D5, E1-E5). Likewise, DNA from *Histophilus somni*, *Mannheimia haemolytica* or *Mycoplasma bovis* could not be detected by PCR in BAL from any animal.

## Discussion

This is the first study that describes bronchoalveolar proteome profiles induced by BRSV in calves, which is considered as a very pertinent model of HRSV in humans. The absence of detectable DNA from bacterial opportunistic pathogens in the animals with the most severe clinical signs of disease and extensive lung lesions highlights the direct pathogenicity of BRSV. The data suggested that neutrophil responses are dominant during primary BRSV infections at the peak of clinical signs, at least at the protein level (detectable by LC-MS/MS) in the lumen of the respiratory tract. Although we cannot exclude that these responses were enhanced by the presence of low numbers of *Pasteurella multocida* in some of the calves in the mildest challenge experiment (calf experiment I), the protein pattern was very similar in the more severely ill calves included in experiment II, in the absence of this bacterium. Many of the proteins associated with BRSV-disease were thus related to neutrophils, such as those in azurophilic granules (*e*.*g*. azurocidin, cathepsin G, elastase, myeloperoxidase, and proteinase-3), the neutrophil-specific surface receptor CD177, major internal neutrophil proteins, S100-A8/A9, antimicrobial proteins (*e*.*g*. lactotransferrin, defensins and cathelicidins), histones and neutrophil chemoattractants (*e*.*g*. S100-A9, hematopoietic cell-specific Lyn-substrate 1 and resisitin) [16, 18–21, 31]. Nevertheless, some of these proteins might also have been derived from epithelial cells or macrophages (*e*.*g*. beta defensins, cathelicidins and histones [16, 22]), or monocytes (*e*.*g*. S100-A9 [31]). S100-A8 and S100-A9 were similarly two of the major proteins detected by proteomics in nasal secretions of HRSV-infected children, admitted for routine HRSV diagnosis [6].

In this study, other BRSV-disease associated proteins probably originated from epithelial cells (folate receptor alpha [23]), possibly due to sloughing of such cells into the respiratory lumen, or from systemic acute phase responses (serum amyloid A, haptoglobin) similarly detected by proteomic analyses of lung tissue from HRSV-infected rats and by ELISA in BRSV-infected calves [5, 32]. We additionally detected proteins related to gluconeogenesis possibly enhanced by a catabolic state generated by cell destruction (L-serine dehydratase/L-threonine deaminase), and to lymphocytes and natural killer cell activation and chemotaxis (hematopoietic cell-specific Lyn-substrate, lymphocyte-specific protein 1 [24, 25]). However, overall, compared to the neutrophil-related proteins, these proteins were less numerous, at least at days 6 to 7 after infection.

Neutrophils probably contribute to the clearance of BRSV infections, by removing virus-infected cells [33], but when present in excessive numbers, they appear to have negative effects [2]. In this study, we demonstrated a positive correlation between the number of neutrophils in alveolar septa or the relative expression level of neutrophil-related proteins and gross pathology, clinical signs of respiratory disease and virus shedding. This is in agreement with recent studies that showed a link between disease severity in HRSV-infected children and BAL neutrophil counts as well as interleukin (IL)-8 and IL-17, which, directly or indirectly, are neutrophil chemoattractants [34, 35]. Moreover, it has been suggested that the causal link between severe HRSV infection at an early age and recurrent wheeze [36] and the increased risk of developing asthma and atopy later in life in these individuals [37, 38] can be explained by a genetic predisposition for neutrophil recruitment and activity, through increased IL-8 and decreased IL-10 responses to infection [2, 39, 40]. Furthermore, the clinical signs of BRSV-infection in cattle, which start on PID 3–5, and peak on PID 6–9 [7, 10, 33, 41], are associated with a pulmonary neutrophil response, which peaks on PID 5–6 in the exudate of alveolar lumina [42] and constitutes the predominant cell type in BAL of non-vaccinated, BRSV-infected calves, on PID 6–8 [7, 33, 43].

The detrimental effect of neutrophils is partly due to their contribution to the physical obstruction of small airways that is central in RSV pathogenesis [33, 42, 44]. In susceptible animals with high levels of BRSV replication, we confirmed the presence of proteins associated with NETs, which agrees with previous findings [30]. It is known that the RSV fusion protein induces NETs through activation of toll like receptor 4, or immunoglobulin-complex activation of Fcγ receptors [45] and that these filamentous DNA containing structures can trap and kill microbes, but additionally adversely increase the viscosity of mucus [46]. Herein, NET-associated proteins were not detected by immunoblotting in non-infected animals, nor in vaccinated calves despite high levels of BRSV-specific IgA in BAL, which potentially induces NET formation through FcalphaRI triggering[9, 47]. However, such triggering requires antigen-antibody complex formation and vaccinated animals had no or low levels of detectable virus in BAL. Interleukins were not identified in any calves, probably because of the low concentration of these proteins in the post mortem BALs. We have previously demonstrated significantly higher concentrations of interferon gamma by ELISA in BAL, concentrated 20 x, from BRSV-infected calves compared to that in controls [7], but did not have enough material to continue these investigations in the present study.

As well as contributing to airway plugging, neutrophils contribute to lung remodeling. The neutrophil-derived serine protease elastase, which in this study most significantly correlated with pathology, causes elastine fiber degradation, induces epithelial to mesenchymal transition (EMT), goblet cell metaplasia and mucus production [48–50]. Elastine fibers are major components of pulmonary extracellular matrix, are essential for lung elasticity and are very difficult to repair [48]. During EMT, pneumocytes are transformed into migratory fibroblast-like cells, which following permanent stimuli can result in lung fibrosis [51]. Herein, BRSV disease correlated negatively with LZTFL1, which inhibits the EMT process [52]. Lung remodeling might thus contribute to the long term clinical consequences sometimes observed after RSV-infections, including clinical signs several weeks after viral clearance (personal observations) and reduced growth [53]. Mucins produced by goblet cells were detected in both experiments, in all groups, but were not significantly associated with disease. However, BAL of several susceptible calves contained large aggregates of mucus that were trapped in the gauze when the BAL was filtered.

On the other hand, numerous histones were associated with disease. These proteins probably derived from NETs, or were released to the extracellular space during RSV-induced cellular apoptosis, or cellular senescence induced by oxidative stress [28, 33, 54]. The oxidative stress in the lungs of animals with BRSV disease was probably high, since antioxidant enzymes appeared either consumed or downregulated, although we cannot exclude the possibility that these proteins were competed out in the LC-MS/MS analysis. Such competition could explain that fewer proteins were detected in animals with BRSV disease. Nevertheless, neutrophils are the most potent producers of reactive oxygen species in the lung and there was a negative correlation between antioxidant enzymes and neutrophil-related proteins. A consumption of antioxidants was previously observed in severe chronic obstructive pulmonary disease (COPD), in which neutrophils similarly play a key role and, against which, antioxidant/redox-modulating therapies are beneficial [55, 56]. Superoxide dismutase (SOD)-1, which correlated negatively with all disease parameters, is produced by a multitude of cells, including hepatocytes and T lymphocytes, and is involved in T cell receptor activation [57].

In conclusion, the results obtained in this paper have identified new pathways to target in order to reduce the excessive pulmonary inflammation in RSV-infected calves and man. Our findings suggest that treatments that mitigate neutrophilic responses or have antioxidant properties could improve RSV-induced clinical signs and pathology, in both the short and long terms. High similarities in RSV pathogenesis make the calf model an important complement in HRSV research.

## Supporting information

S1 Table Clinical scoring. Scoring system for respiratory signs of disease in calves.
(DOCX)

S2 Table Proteins names. Names of proteins identified by LC-MS/MS in bronchoalveolar lavage as described in Fig 1.
(XLSX)

S3 Table Proteins expressed at significantly lower levels in BRSV-infected animals compared to in their controls. (XLSX)

S4 Table Correlations between neutrophil-related and antioxidant proteins. (TXT)
