# Supplementary material for: Proteome analysis of bronchoalveolar lavage from calves infected with bovine respiratory syncytial virus—Insights in pathogenesis and perspectives for new treatments
Source: PLoS One. 2017 Oct 16;12(10):e0186594. doi: 10.1371/journal.pone.0186594 (PMC5643112; doi:10.1371/journal.pone.0186594)
Supplement: S1 Table — Scoring system for respiratory signs of disease in calves. (DOCX) [file pone.0186594.s001.docx]

Clinical scoring

| *Score* | *Fever*  *(C°)* | *Cough* | *Auscultation noise* | *Nasal discharge* | *Tachypnea*  *(breaths/min)* |
| --- | --- | --- | --- | --- | --- |
| 0 | ≤ 39,5 | no cough observed | no abnormal sounds noticed upon lung auscultation | normal nasal discharge | <49 |
| 1 | 39,6-39,9 | only cough on compression of trachea | wheezing sounds noticed  upon lung auscultation | serous and/or very little mucopurulent nasal discharge | 50-54 |
| 2 | 40-40,4 | spontaneous cough during 20 min observation |  | moderate mucopurulent nasal discharge | 55-64 |
| 3 | 40,5-40,9 |  |  | marked mucopurulent nasal discharge | 65-74 |
| 4 | >40,9 |  |  |  | 75-85 |
